# Latent Subtype of Cognitive Frailty among Multimorbidity Older Adults and Their Association with Social Relationships

**DOI:** 10.3390/healthcare11131933

**Published:** 2023-07-04

**Authors:** Dandan Jiao, Xiang Li, Zhu Zhu, Jinrui Zhang, Yang Liu, Mingyu Cui, Munenori Matsumoto, Alpona Afsari Banu, Yuko Sawada, Taeko Watanabe, Emiko Tanaka, Tokie Anme

**Affiliations:** 1 Department of Nursing, The First Affiliated Hospital, and College of Clinical Medicine, Henan University of Science and Technology, Luoyang 471003, China; 2 School of Comprehensive Human Science, University of Tsukuba, Tsukuba 305-8577, Japan; 3 Department of Physical Therapy, Morinomiya University of Medical Sciences, Osaka 559-0034, Japan; 4 Faculty of Nursing, Shukutoku University, Chiba 260-8701, Japan; 5 Faculty of Nursing, Musashino University, Tokyo 135-8181, Japan; 6 Faculty of Medicine, University of Tsukuba, Tsukuba 305-8575, Japan

**Keywords:** cognitive frailty, social relationship, latent class analysis, functioning

## Abstract

This study aimed to explore all the relevant subtypes of cognitive frailty among Japanese community-dwelling older adults with multimorbidity. Moreover, it examined the associations between these potential subtypes of cognitive frailty and social relationships. This study targeted relevant cross-sectional data regarding community-based older adults with multimorbidity. It employed a person-centered method to perform a latent class analysis and explore the subtypes of cognitive frailty among older adults. Moreover, a multinominal logistic regression analysis was employed to examine the association between potential subtypes of cognitive frailty and social relationships. Data for 396 participants (mean age, 75.8 [SD, 7.3] years; 51.3% females) were analyzed. Three cognitive frailty subtypes were subsequently revealed: the robust group (42.0%), the group with partial cognitive frailty (38.6%), and the group with cognitive frailty (19.4%). People with high levels of social relationships were more likely to be in the robust and the partial cognitive frailty groups. This study identified different subtypes of cognitive frailty among multimorbid older adults and highlighted the significance of social relationships. These findings could serve as a reference for conceptualizing cognitive frailty through the person-centered method. Promoting a high level of social relationships could be useful to prevent the cognitive frailty among older adults with multimorbidity.

## 1. Introduction

The world is witnessing rapid population aging; the number of people aged 65 years and above is expected to rise to one out of six by 2050 [1]. Along with aging, increasing numbers of older adults are experiencing multimorbidity, which is defined as the simultaneous experience of more than two chronic diseases [2] by one person. The increased likelihood of multimorbidity has been related to unhealthy lifestyle behaviors (e.g., smoking and abnormal body mass index) [3].

Several studies have linked multimorbidity to a high probability of adverse health outcomes among older adults, including physical limitations [4,5,6], problems in both basic activities and instrumental activities of daily living [7,8], and mortality [9]. The effects of multimorbidity on cognition function have received relatively little exploration [10]. However, recently, evidence has been emerging in this regard; for example, a cross-sectional study using global aging and adult health-related data indicated that multimorbidity was significantly associated with higher chances of experiencing cognitive impairment [11]. Subsequent longitudinal studies also supported this association. A 14-year longitudinal study indicated that multimorbidity was associated with a steep cognition decline [10]. Similarly, other studies also indicated significant associations between multimorbidity and a cognitive decline [12,13] as well as subjective cognitive complaints [14]. Further, a systematic review indicated that there was a higher prevalence of cognitive complaints among those with multimorbidity [15]. However, these studies have tended to examine the effects of multimorbidity on physical function and cognitive function separately, while in actuality, a physical decline and cognitive decline may often co-exist normally [16]. On the other hand, such research has widely shown an interest in the simultaneous presence of physical frailty and cognitive impairment, which has been considered as “cognitive frailty” [17]. Among community-dwelling older adults, the prevalence of cognitive frailty ranges from 9–21.8% [18,19]. Cognitive frailty is much more likely to predict adverse health outcomes such as fall-related fractures [20] and disability and death [21,22].

Multimorbidity has been associated with adverse health outcomes, and cognitive frailty has been independently related to stressful outcomes as well. The simultaneous occurrence of both can worsen affected individuals’ health conditions. Therefore, it is important to make more efforts toward exploring various factors related to the prevention of cognitive function deterioration among older multimorbid adults.

A lack of social relationships has been associated with cognitive frailty among older people in general [23]. In terms of the association between social relationships and cognitive/physical frailty, certain evidence has been documented. For example, a 10-year longitudinal study showed that instrumental or emotional social support could reduce the physical frailty among older people [24]. Social participation could also predict a probability of frailty improvement [25]. In addition, less social relationships increased the risk of physical frailty deterioration in older people [26]. However, previous studies on the link between social relationships and cognitive/physical frailty are separate, which means the outcome is either cognitive frailty or physical frailty. Few studies examined the combined outcome of both physical and cognitive frailty. Wang et al. [27] examined the association between social support and cognitive frailty among older Chinese people and proved that lower social relationships are also linked to the increased risk of cognitive frailty. Wang and colleague’s study mainly focused on general older people. The protective role of social relationships on frailty remains unknown among people with chronic disease, especially multimorbid older people. Therefore, the current study aimed to examine the association between social relationships and cognitive frailty (simultaneous physical and cognitive frailty).

On the other hand, to date, though the term “cognitive frailty” has been widely accepted in research and practice, no consensus has been achieved on the definition of and tools for assessing cognitive frailty [17,19]. This study employed a person-centered approach to assess various subtypes of cognitive frailty. A latent class analysis (LCA) is a person-centered approach that allows for the clustering of the subtypes of latent variables in order to consider the heterogeneity of older people [28]. Majnarić et al. [29] performed an LCA analysis and identified four subtypes of cognitive frailty; however, they did not focus on participants with multimorbidity and did not explore the influence of social relationships on the identified subtypes. To date, few empirical studies have used the person-centered method to explore cognitive subtypes.

Considering such research gaps, we employed the LCA method to explore the cognitive frailty status of community-dwelling older adults with multimorbidity and examined how their social relationships were linked to the identified subtypes. A definite identification of the profile of cognitive frailty could contribute to the ongoing argument regarding the conceptualization of cognitive frailty. Further, this study could also provide evidence for future interventions regarding cognitive frailty.

We hypothesized that different cognitive frailty subtypes exist, and high social relationships are associated with less cognitive frailty among multimorbid older adults.

## 2. Materials and Methods

### 2.1. Design

This study derived data from wave 2017 from an ongoing cohort project. The relevant data were collected from 1 April to 15 May 2017. The project, which was started in 1991, is conducted in a suburban area of central Japan. This project was commenced with the aim of identifying and exploring factors contributing to health and longevity as well as the well-being of the local residents. The survey is conducted every 3 years, and its questionnaires are mailed to all the residents of the selected research area.

### 2.2. Participants

To avoid selection bias, all the residents, from children to older people who were living in the suburban area T, were enrolled. In 2017, the total number of older people aged 65 or over was 1301, and 1088 of them responded to the survey (response rate, 83.6%). The study participants were older adults, and the inclusion criteria were as follows: (1) dementia-free multimorbid individuals aged 65 years or above; (2) individuals who provided data on at least one item of the cognitive indicator. Participants with incomplete missing data regarding the cognitive indicators were excluded. Based on the criteria, 418 participants with multimorbidity were targeted for the (current) study. From this targeted population, 396 were included in the final analysis, while 20 participants with dementia and 2 participants with completely missing data regarding the cognitive frailty indicator were excluded. The examined chronic conditions included hypertension, stroke, heart disease, diabetes, hyperlipidemia, lung disease, stomach/liver/gallbladder disorders, kidney disorders, musculoskeletal disorders, cancer, immune disease, depression, Parkinson’s disease, and eye and ear disorders. Multimorbidity was indicated when the participants answered affirmatively regarding having any two or more of these diseases.

### 2.3. Measures

#### 2.3.1. Cognitive Frailty Indicators

Physical and cognitive indicators were extracted from the Kihon Checklist, which is widely used to identify individuals who are at risk of needing long-term support or care and who can qualify for the “frailty status” in Japan [30]. The current study utilized the physical and cognitive function domains and included both physical and cognitive functions as a useful approach for predicting frailty among older adults [30]. Each item was scored as “good” if 0 was scored and “poor” if 1 was scored. The Cronbach alpha for physical and cognitive function is 0.713 and 0.676, respectively.

#### 2.3.2. Social Relationships

This current study assessed social relationships using the Index of Social Interaction (ISI) [31]. The ISI, which includes 18 items and has a good validity and reliability (Cronbach alpha, 0.78), measures various aspects of social relationships in daily settings. These include independence, social curiosity, interaction, societal participation, and feeling safe. Independence includes four items: evaluating an individual’s motivation to live an active life, taking an active approach toward life, motivation to live a healthy life, and practicing a regular lifestyle. Social curiosity evaluates individuals’ reading of newspapers/books, trying of new equipment, practice of a hobby, and feelings of importance (total: five items). Interaction, which includes three items, assesses individuals’ communication with their family members/non-family members and any interactions with non-family members. Societal participation, which includes four items, assesses individuals’ participation in social groups/neighborhood affairs, their television watching habits, and their proactive role in society. Feelings of safety, which include two items, evaluate whether individuals have someone they can turn to for counsel and for the provision of support in any emergent situations. For each item, a “rare” or “no” response was coded as 0, and any other response was coded as 1 point. The total score summed up all the items’ scores, ranging from 0 to 18. A higher score indicated a high level of social relationships. The total score was used and treated as a continuous variable in the analysis.

#### 2.3.3. Covariables

Age, sex, living status, exercise, long-term care needs, and smoking/drinking behaviors were taken as covariates. Age was entered as a continuous variable into this analysis. Living status was coded as “alone” or “not alone”. Exercise was coded as “not doing” when respondents answered “no” and “doing” when the responses were “always” or “often”. The long-term care status was assessed using the question “Do you need support or care in your daily life?”, and the responses were dichotomized as “no need” for no responses and “need” otherwise. For the evaluation of the smoking and drinking status, the questions “Do you smoke?” and “Do you drink?” were utilized. The answers were coded as “smoking” if the participants responded “everyday” or “sometimes” and “not smoking” if they responded “smoke before but stopped now” or “do not smoke”. Similarly, the evaluation of the drinking status adopted the same coding method; “drinker” was coded if the response was “everyday” or “sometimes”, and “non-drinker” was coded if the response was “almost do not drink” or “do not drink”.

### 2.4. Statistical Analysis

Various cognitive frailty subtypes were explored using the LCA method. The numbers of the latent patterns were estimated by comparing the indicators of the Akaike information criterion (AIC), Bayesian information criterion (BIC), and sample-adjusted Bayesian information criterion (aBIC). Smaller values predicted a better model fit [32]. Some other model fitting considerations included entropy and the Lo-Mendell-Rubin (LMR) test. Entropy was used to assess the model’s accuracy within a range of 0 to 1; a higher score predicted better model goodness. The entropy values of 0.8 and 0.6 were regarded as indicating high and medium accuracy, respectively [33]. The p-values of the Lo-Mendell-Rubin (LMR) test and bootstrap likelihood ratio tests (BLRT) were used for estimating whether the k class was better than the k-1 class. Participants having incomplete data regarding all the cognitive indicators were excluded. Full-information maximum likelihood estimation was used in the LCA in order to deal with the missing data.

Second, Chi square/Mann–Whitney U tests were performed to examine the association between demographic characteristics and the class memberships related to cognitive frailty for categorical and numerical data, respectively.

Finally, this study employed a multi-nominal logistic regression analysis to form associations between ISI and the cognitive frailty patterns after controlling for confounding factors that showed statistical significance in the Chi square/Mann–Whitney U tests.

The LCA analysis was conducted in Mplus (Mplus Version 8.0) (Muthén and Muthén, Los Angeles, CA, USA). SPSS 27.0 (SPSS Inc, Chicago, IL, USA) was used for performing the univariate and regression analysis. The *p*-value of 0.05 was set as statistical significance.

### 2.5. Ethical Considerations

This study was approved by the Ethics Committee of the University of which one of the authors is affiliated (No. 1331-4). This study was performed in line with the Ethical Principles for Medical Research Involving Human Subjects stated in the Declaration of Helsinki. Participants’ written informed consent was waived in this study because anonymous data were provided by the study area and participants reserved their right to opt out from the research according to the Japanese Ethical Guidelines for Medical and Health Research Involving Human Subjects.

## 3. Results

Table 1 shows the demographic information of the study participants; 396 were included in the current analysis. The mean age of the participants was 75.8 ± 7. 3. Over half of the participants were female, not living alone, non-drinkers or non-smokers, had no need for long-term care, and were exercisers. Our sample had relatively few cases of individuals who smoked—only 25 cases in the total sample. Therefore, the smoking category was integrated into the drinking category.

The model fitting information of the LCA showed that class 3 had relatively smaller values like those of AIC, BIC, and aBIC (Table 2). It should be noted that the best model cannot be identified with a single indicator. Thus, we chose three class models after considering all the indicators, parsimony and model interpretability [34], and the rule of thumb that at least 5% of the sample should be included in any identified subtype [33].

**Figure 1:**
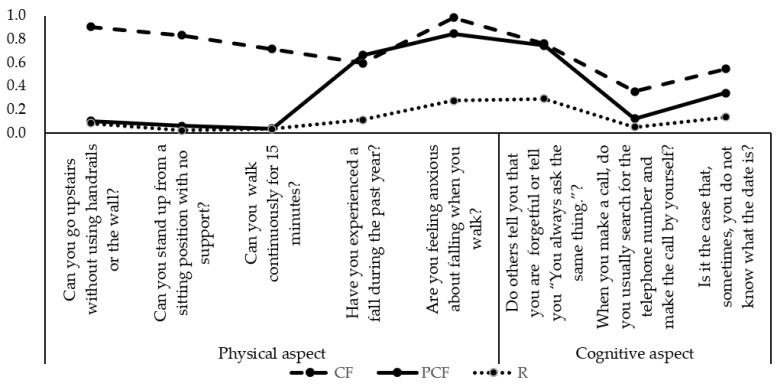
Item probability plot for the class membership of cognitive frailty.

Table 3 and Figure 1 present the conditional probabilities of cognitive frailty for the identified three latent classes. The identified first class, which showed a high conditional probability of poor responses for all the indicators, was labeled as the cognitive frailty (CF) class, showing poor performance in all aspects (climbing upstairs, walking, standing, falling and fear of falling, memory function). The second group showed a moderately high conditional probability of poor responses and was labeled as the partial cognitive frailty (PCF) class, especially in falling, fear of falling, and memory aspects. The third class showed the lowest probability of poor responses to all items and was labeled as the robust (R) class. The class membership probabilities were CF, 19.4% (*n* = 77); PCF, 38.6% (*n* = 153); and R, 42.0% (*n* = 166).

Table 4 shows the demographic characteristics of the three latent classes of frailty. Age, sex, drinking/smoking behavior, exercise, long-term care need, and ISI were associated with the class membership of cognitive frailty. Compared to the other robust and prefrailty groups, the CF group was older, had female individuals, individuals with a need for long-term care support, individuals who did not exercise, a higher proportion of no smoking/no drinking behavior, individuals with fewer social relationships, and individuals having more disease than the R and PCF groups. Further, the living status in the frailty group was higher than that in the R group but lower than that in the PCF group.

Table 5 shows that the individuals who experienced a one-point increase in the index of social relationships were more likely to be in the R (OR = 1.40, 95% CI = 1.15–1.70) or PCF groups (OR = 1.28, 95% CI = 1.08–1.57) after adjusting for other covariates.

## 4. Discussion

The current study explored the subtypes of cognitive frailty among multimorbid participants and their association with social relationships. We found three main subtypes: robust, partial cognitive frailty, and cognitive frailty; furthermore, participants with high numbers of social relationships were more likely to be in the robust and partial cognitive frailty groups rather than the cognitive frailty group.

This study found that the prevalence of cognitive frailty among multimorbid community-dwelling older adults was 19.4%; this figure is similar to that among participants with chronic diseases. One systematic review evaluated and found that cognitive impairment among people with chronic kidney disease was 21.5% [35]. Similarly, older people with heart failure showed a 23.0% chance of developing cognitive frailty [36]. In past studies, the prevalence of cognitive frailty was lower than that in our study, which focused on multimorbid older adults rather than the general population, with a prevalence ranging from 2.1% to 7.2% [37,38]. We could attribute the higher prevalence of cognitive frailty in our study to the inclusion of multimorbid participants because multimorbidity is a risk factor for cognitive frailty [37]. Thus, cognitive frailty is more common among multimorbid older adults compared to the general population of older adults.

This study used the LCA method to identify three latent classes of cognitive frailty among multimorbid older adults. Bekić et al. [39] explored four latent classes of cognitive frailty: the high function group, the cognitive impairment group, the cognitive frailty group, and the physical frailty group; these groups included older adults aged 60 years or above [39]. The differences in the classes may depend on the differences between the cognitive indicators in our study and those in Bekić et al.’s [40] study. Another reason for the different numbers of identified classes may be the participants’ heterogeneity; our study included older adults with multimorbidity, while their study did not do so. The evidence on cognitive frailty among multimorbid individuals has been very limited. This current study’s findings aim to fill this research gap. Moreover, use of the LCA method can serve as a supplementary strategy alongside cognitive conceptualization because, currently, the conceptualization and measurement of cognitive frailty is often inconsistent.

This study suggested that a high number of social relationships was associated with inclusion in the robust or partial cognitive frailty groups compared to cognitive frailty among older multimorbid adults. This finding agrees with the existing evidence. A previous systematic review revealed that social relationships can be beneficial to older adults’ cognitive function [40]. Better social participation acts as a protective factor against cognitive frailty [38]. Likewise, a high level of social interaction predicted a lower probability of cognitive frailty among older adults with hypertension in comparison to those with low levels of social support [41]. The study result showed that among multimorbid older adults, social relationships could also play a protective role. The results added a piece of evidence to the current research that both for general older people and older people with chronic disease, social relationships are beneficial to their cognitive frailty function.

Multimorbidity and frailty are two common geriatric symptoms among older people. Multimorbidity is associated with physical frailty [42] as well as cognitive impairment [10]. The current study confirmed the co-existing physical and cognitive impairments among multimorbid older adults. The co-existent frailty impairments result in a further adverse outcome among older multimorbid adults. Therefore, strategies should be taken to break the vicious loop. The current study results suggest a promising intervention strategy for managing multimorbidity because cognitive frailty can be reversible [43]. This study’s identification of the beneficial influence of social relationships could inform the direction of interventional studies for preventing or decreasing cognitive frailty among multimorbid older adults. Possible pathways for linking social relationships to health outcomes might provide a buffering effect from social relationships. It has been noted that, when people are facing a stressful situation, supportive and caring interactions can act as a buffer to preserve cognitive function [44]. It is therefore reasonable that, while multimorbidity may be a stressful health situation, a high number of social relationships could serve as a buffer against cognitive frailty and link multimorbid individuals to robust or partial cognitive frailty conditions.

Our study’s main strength was that it focused on multimorbid participants and their cognitive frailty, a subject that has not received much examination. Our analysis employed the LCA method to explore the cognitive frailty subtypes that could supplement the current definition of cognitive frailty, considering the ongoing debate on definitions for cognitive frailty. Further, we recognized that linking social relationships to cognitive frailty among multimorbid older adults may be useful for informing multimorbidity management strategies—a finding that could be useful for clinicians, community health workers, and related health professionals. Finally, this study also examined various aspects of social relationships in the daily life of multimorbid older adults.

Despite the relative strength of its findings, this study had several limitations. First, this study produced cross-sectional results and thus could not deduce the causal effects of social relationships on cognitive frailty. Second, it used a cognitive indicator that mainly evaluates memory function due to the data availability of the dataset. More domain indicators (e.g., attention, perception, and so on) could be included in future studies. Third, even the self-report assessments of a physical aspect were comparable to the objective measures, e.g., time up and go test, and results from a clinical physical examination would be more reliable. Fourth, focusing on a longitudinal latent transition analysis, which represents the progress of cognitive frailty and how social relationships can be associated with the progress of cognitive frailty changing, would provide more interesting results. Fifth, our study did not examine the bidirectional impact between cognitive frailty and multimorbidity; future studies are suggested to elucidate their interplay for identifying the possible double trouble for improving health longevity. Sixth, a more precise three-step method [45] could be employed for a latent variable analysis in a future study.

## 5. Conclusions

The association between social relationships and better cognitive function could aid health professionals in evaluating their social conditions and any possible cognitive function outcomes. In this way, a supportive system that includes social relationships and prevents adverse cognitive functions can be created for community-dwelling multimorbid older adults.

## Figures and Tables

**Table 1:** Demographic information of the participants (N = 396).

Items	Categories	*n*	%
Age	Mean (±SD)	75.8 ± 7.3	
	Missing	0	0.0
Sex	Male	193	48.7
	Female	203	51.3
	Missing	0	0.0
Living status	Alone	23	5.8
	Not alone	341	86.1
	Missing	32	8.1
Drinking/Smoking	Yes	139	35.1
	No	249	62.9
	Missing	8	2
Long term care	Need	88	22.2
	No need	308	77.8
	Missing	0	0.0
Exercise	Doing	212	53.5
	Not doing	150	37.9
	Missing	34	8.6
Chronic disease	Mean (±SD)	2.6 ± 0.8	
	Missing	0	0.0
ISI	Mean (±SD)	16.3 (± 2.2)	
	Missing	29	7.3

ISI: index of social relationships.

**Table 2:** Model fitting information of the LCA models.

Models	AIC	BIC	ABIC	Entropy	LMR	BLRT
Class 1	3451.665	3483.516	3458.132			
Class 2	3077.052	3144.737	3090.795	0.871	<0.001	<0.001
Class 3	3029.740	3133.311	3050.812	0.683	<0.001	<0.001
Class 4	3028.503	3167.852	3056.797	0.726	0.046	0.192
Class 5	3024.62	3199.802	3060.190	0.765	0.121	0.136
Class 6	3027.678	3238.693	3070.523	0.754	0.582	1.000

AIC: Akaike information criteria; BIC: Bayesian information criteria; aBIC: adjusted Bayesian information criteria; LMR: Lo-Mendell-Rubin test; BLRT: bootstrapped likelihood ratio tests.

**Table 3:** Conditional item response probability of cognitive frailty.

Items	Class 1 CF	Class 2 PCF	Class 3 R
Can you go upstairs without using handrails or the wall?	0.906	0.103	0.084
Can you stand up from a sitting position with no support?	0.835	0.061	0.022
Can you walk continuously for 15 min?	0.715	0.038	0.033
Have you experienced a fall during the past year?	0.594	0.663	0.114
Are you feeling anxious about falling when you walk?	0.983	0.848	0.275
Do others tell you that you are forgetful or tell you that “You always ask the same thing”?	0.763	0.745	0.292
When you make a call, do you usually search for the telephone number and make the call by yourself?	0.353	0.122	0.051
Is it the case that, sometimes, you do not know what the date is?	0.548	0.342	0.135

CF: cognitive frailty, PCF: partial cognitive frailty, R: robust.

**Table 4:** Association between demographic information and the class memberships of cognitive frailty.

Items	Categories	Class 3: R (*n* = 166)		Class 2: PCF (*n* = 153)		Class 1: CF (*n* = 77)		z/χ²	*p*
		*n*	%	*n*	%	*n*	%		
Age	Mean (±SD)	73.0 (±6.1)		75.5 (±7.4)		82.9 (±7.2)		68.573	<0.001
Sex	Male	95	57.2	73	47.7	25	32.5	13.013	<0.001
	Female	71	42.8	80	52.3	52	67.5		
Drinking or Smoking	Yes	71	43.3	59	39.9	9	11.8	24.042	<0.001
	No	93	56.7	89	60.1	67	88.2		
Long-term care need	Need	10	6	29	19	49	63.6	102.554	<0.001
	No need	156	94	124	81	28	36.4		
Exercise	Doing	109	70.3	80	57.6	23	33.8	26.042	<0.001
	Not doing	46	29.7	59	42.4	45	66.2		
Living status	Alone	6	3.9	12	8.6	5	6.8	2.736	0.255
	Not alone	146	96.1	127	91.4	68	93.2		
Chronic disease	Mean (±SD)	2.4 (±0.7)		2.6 (±0.8)		2.8 (±1.0)		13.848	<0.001
ISI	Mean (±SD)	16.9 (±1.5)		16.5 (±2.0)		14.6 (±2.7)		56.224	<0.001

CF: cognitive frailty, PCF: partial cognitive frailty, R: robust, ISI: index of social interaction.

**Table 5:** Multinomial logistic regression analysis for the class memberships of cognitive frailty.

Items	Category	Class 2: PCF				Class 3: R	
		OR	95% CI	*p*	OR	95% CI	*p*
ISI		1.28	1.08–1.57	0.004	1.40	1.15–1.70	<0.001
Age		0.93	0.88–0.95	0.020	0.89	0.83–0.94	<0.001
Sex	Male	3.34	1.40–7.98	0.007	6.11	2.41–15.45	<0.001
	Female	Ref.					
Drinking or Smoking	No	0.36	0.12–1.04	0.059	0.28	0.07–1.19	0.086
	Yes	Ref.					
Exercise	Do	2.78	1.25–6.25	0.013	4.76	2.02–12.50	<0.001
	Do not	Ref.					
Long-term care	No need	5.91	2.49–14.03	<0.001	14.61	5.20–41.11	<0.001
	Need	Ref.					

PCF: partial cognitive frailty; R: robust; reference group = class 1; CF: cognitive frailty; ISI: index of social interaction. ISI and age are continuous variables.

## Data Availability

Not applicable.

## References

[1] United Nations (2019). World Population Ageing 2019: Highlights. https://www.un.org/en/development/desa/population/publications/pdf/ageing/WorldPopulationAgeing2019-Highlights.pdf.

[2] World Health Organization (2016). Multimorbidity: Technical Series on Safer Primary Care.

[3] Fortin M., Haggerty J., Almirall J., Bouhali T., Sasseville M., Lemieux M. (2014). Lifestyle factors and multimorbidity: A cross sectional study. BMC Public Health.

[4] Jiao D., Watanabe K., Sawada Y., Tanaka E., Watanabe T., Tomisaki E., Ito S., Okumura R., Kawasaki Y., Anme T. (2021). Multimorbidity and functional limitation: The role of social relationships. Arch. Gerontol. Geriatr..

[5] Lange-Maia B.S., Karvonen-Gutierrez C.A., Kazlauskaite R., Strotmeyer E.S., Karavolos K., Appelhans B.M., Janssen I., Avery E.F., Dugan S.A., Kravitz H.M. (2020). Impact of Chronic Medical Condition Development on Longitudinal Physical Function from Mid- to Early Late-Life: The Study of Women’s Health Across the Nation. J. Gerontol. A Biol. Sci. Med. Sci..

[6] Quiñones A.R., Markwardt S., Botoseneanu A. (2016). Multimorbidity combinations and disability in older adults. J. Gerontol. A Biol. Sci. Med. Sci..

[7] Schmidt T.P., Wagner K.J.P., Schneider I.J.C., Danielewicz A.L. (2020). Multimorbidity patterns and functional disability in elderly Brazilians: A cross-sectional study with data from the Brazilian National Health Survey. Cad. Saúde Pública.

[8] Williams J.S., Egede L.E. (2016). The association between multimorbidity and quality of life, health status and functional disability. Am. J. Med. Sci..

[9] Nguyen H., Wu Y.T., Dregan A., Vitoratou S., Chua K.C., Prina A.M. (2020). Multimorbidity patterns, all-cause mortality and healthy aging in older English adults: Results from the English Longitudinal Study of Aging. Geriatr. Gerontol. Int..

[10] Wei M.Y., Levine D.A., Zahodne L.B., Kabeto M.U., Langa K.M. (2020). Multimorbidity and cognitive decline over 14 years in older Americans. J. Gerontol. A Biol. Sci. Med. Sci..

[11] Koyanagi A., Smith L., Shin J.I., Oh H., Kostev K., Jacob L., Abduljabbar A.S., Haro J.M. (2021). Multimorbidity and Subjective Cognitive Complaints: Findings from 48 Low- and Middle-Income Countries of the World Health Survey 2002–2004. J. Alzheimer’s Dis..

[12] Kao S.L., Wang J.H., Chen S.C., Li Y.Y., Yang Y.L., Lo R.Y. (2021). Impact of Comorbidity Burden on Cognitive Decline: A Prospective Cohort Study of Older Adults with Dementia. Dement. Geriatr. Cogn. Disord..

[13] Lee Y., Cho C.C. (2021). Examining the effects of multiple chronic conditions on cognitive decline and potential moderators among older Koreans: Findings from the Korean Longitudinal Study of Ageing 2006–2016. Arch. Gerontol. Geriatr..

[14] Jacob L., Haro J.M., Koyanagi A. (2019). Physical Multimorbidity and Subjective Cognitive Complaints among Adults in the United Kingdom: A Cross-sectional Community-based Study. Sci Rep..

[15] Hill N.L., Bhargava S., Brown M.J., Kim H., Bhang I., Mullin K., Phillips K., Mogle J. (2021). Cognitive complaints in age-related chronic conditions: A systematic review. PLoS ONE.

[16] Morley J.E., Morris J.C., Berg-Weger M., Borson S., Carpenter B.D., Del Campo N., Dubois B., Fargo K., Fitten L.J., Flaherty J.H. (2015). Brain health: The importance of recognizing cognitive impairment: An IAGG consensus conference. J. Am. Med. Dir. Assoc..

[17] Sugimoto T., Arai H., Sakurai T. (2021). An update on cognitive frailty: Its definition, impact, associated factors and underlying mechanisms, and interventions. Geriatr. Gerontol. Int..

[18] Das S. (2022). Cognitive frailty among community-dwelling rural elderly population of West Bengal in India. Asian J. Psychiatry.

[19] Qiu Y., Li G., Wang X., Zheng L., Wang C., Wang C., Chen L. (2022). Prevalence of cognitive frailty among community-dwelling older adults: A systematic review and meta-analysis. Int. J. Nurs. Stud..

[20] Tsutsumimoto K., Doi T., Makizako H., Hotta R., Nakakubo S., Makino K., Suzuki T., Shimada H. (2018). Cognitive frailty is associated with fall-related fracture among older people. J. Nutr. Health Aging.

[21] Chen C., Park J., Wu C., Xue Q., Agogo G., Han L., Hoogendijk E.O., Liu Z., Wu Z. (2020). Cognitive frailty in relation to adverse health outcomes independent of multimorbidity: Results from the China health and retirement longitudinal study. Aging.

[22] Lee W.J., Peng L.N., Liang C.K., Loh C.H., Chen L.K. (2018). Cognitive frailty predicting all-cause mortality among community-living older adults in Taiwan: A 4-year nationwide population-based cohort study. PLoS ONE.

[23] Malek Rivan N.F., Shahar S., Rajab N.F., Singh D.K.A., Din N.C., Hazlina M., Hamid T.A.T.A. (2019). Cognitive frailty among Malaysian older adults: Baseline findings from the LRGS TUA cohort study. Clin. Interv. Aging.

[24] Chu W.M., Tange C., Nishita Y., Tomida M., Shimokata H., Otsuka R., Lee M.C., Arai H. (2023). Effect of different types of social support on physical frailty development among community-dwelling older adults in Japan: Evidence from a 10-year population-based cohort study. Arch. Gerontol. Geriatr..

[25] Ye B., Chen H., Huang L., Ruan Y., Qi S., Guo Y., Huang Z., Sun S., Chen X., Shi Y. (2020). Changes in frailty among community-dwelling Chinese older adults and its predictors: Evidence from a two-year longitudinal study. BMC Geriatr..

[26] Park J., Shin H.E., Kim M., Won C.W., Song Y.M. (2023). Longitudinal association between eating alone and deterioration in frailty status: The Korean Frailty and Aging Cohort Study. Exp. Gerontol..

[27] Wang Y., Li J., Fu P., Jing Z., Zhao D., Zhou C. (2022). Social support and subsequent cognitive frailty during a 1-year follow-up of older people: The mediating role of psychological distress. BMC Geriatr..

[28] Muthén B., Muthén L.K. (2000). Integrating person-centered and variable-centered analyses: Growth mixture modeling with latent trajectory classes. Alcohol Clin. Exp. Res..

[29] Majnarić L.T., Bekić S., Babič F., Pusztová Ľ., Paralič J. (2020). Cluster analysis of the associations among physical frailty, cognitive impairment and mental disorders. Med. Sci. Monit..

[30] Fukutomi E., Okumiya K., Wada T., Sakamoto R., Ishimoto Y., Kimura Y., Kasahara Y., Chen W.L., Imai H., Fujisawa M. (2013). Importance of cognitive assessment as part of the “Kihon Checklist” developed by the Japanese Ministry of Health, Labor and Welfare for prediction of frailty at a 2-year follow up. Geriatr. Gerontol. Int..

[31] Anme T. (1997). Evaluation of environmental stimulation and its relation to physical deterioration in the elderly after 3 years--A health-social longitudinal study. Nihon Koshu Eisei Zasshi.

[32] Muthén B., Asparouhov T. (2002). Latent variable analysis with categorical outcomes: Multiple-group and growth modeling in Mplus. Mplus Web Notes.

[33] Cheng G.H.L., Sung P., Chan A., Ma S., Malhotra R. (2022). Transitions between social network profiles and their relation with all-cause mortality among older adults. Soc. Sci. Med..

[34] Collins L.M., Lanza S.T. (2010). Latent Class and Latent Transition Analysis: With Applications in the Social, Behavioral, and Health Sciences.

[35] Shen Z., Ruan Q., Yu Z., Sun Z. (2016). Chronic kidney disease-related physical frailty and cognitive impairment: A systemic review. Geriatr. Gerontol. Int..

[36] Yamamoto S., Yamasaki S., Higuchi S., Kamiya K., Saito H., Saito K., Ogasahara Y., Maekawa E., Konishi M., Kitai T. (2022). Prevalence and prognostic impact of cognitive frailty in elderly patients with heart failure: Sub-analysis of FRAGILE-HF. ESC Heart Fail..

[37] Kim H., Awata S., Watanabe Y., Kojima N., Osuka Y., Motokawa K., Sakuma N., Inagaki H., Edahiro A., Hosoi E. (2019). Cognitive frailty in community-dwelling older Japanese people: Prevalence and its association with falls. Geriatr. Gerontol. Int..

[38] Xie B., Ma C., Chen Y., Wang J. (2021). Prevalence and risk factors of the co-occurrence of physical frailty and cognitive impairment in Chinese community-dwelling older adults. Health Soc. Care Community.

[39] Bekić S., Babič F., Pavlišková V., Paralič J., Wittlinger T., Majnarić L.T. (2021). Clusters of physical frailty and cognitive impairment and their associated comorbidities in older primary care patients. Healthcare.

[40] Kelly M.E., Duff H., Kelly S., McHugh Power J.E., Brennan S., Lawlor B.A., Loughrey D.G. (2017). The impact of social activities, social networks, social support and social relationships on the cognitive functioning of healthy older adults: A systematic review. Syst. Rev..

[41] Wang C., Zhang J., Hu C., Wang Y. (2021). Prevalence and Risk Factors for Cognitive Frailty in Aging Hypertensive Patients in China. Brain Sci..

[42] Vetrano D.L., Palmer K., Marengoni A., Marzetti E., Lattanzio F., Roller-Wirnsberger R., Lopez Samaniego L., Rodríguez-Mañas L., Bernabei R., Onder G. (2019). Frailty and multimorbidity: A systematic review and meta-analysis. J. Gerontol. Ser. A.

[43] Facal D., Maseda A., Pereiro A.X., Gandoy-Crego M., Lorenzo-López L., Yanguas J., Millán-Calenti J.C. (2019). Cognitive frailty: A conceptual systematic review and an operational proposal for future research. Maturitas.

[44] Berkman F.L., Kawachi I., Glymour M.M. (2014). Social Epidemiology.

[45] Asparouhov T., Muthén B. (2014). Auxiliary variables in mixture modeling: Three-step approaches using Mplus. Struct. Equ. Model..

